# Supplementary material for: 3D Cell Culture of Human Salivary Glands Using Nature-Inspired Functional Biomaterials: The Egg Yolk Plasma and Egg White
Source: Materials (Basel). 2020 Oct 28;13(21):4807. doi: 10.3390/ma13214807 (PMC7672643; doi:10.3390/ma13214807)
Supplement: Supplementary file 1 [file materials-13-04807-s001.zip › Figure S10 Videos/Read Me.rtf]

We were able to open the videos using VLC player on mac. 
